# Self-Assembly of Tail Tube Protein of Bacteriophage vB_EcoS_NBD2 into Extremely Long Polytubes in *E. coli* and *S. cerevisiae*

**DOI:** 10.3390/v11030208

**Published:** 2019-03-01

**Authors:** Aliona Špakova, Eugenijus Šimoliūnas, Raminta Batiuškaitė, Simonas Pajeda, Rolandas Meškys, Rasa Petraitytė-Burneikienė

**Affiliations:** 1 Institute of Biotechnology, Life Sciences Center, Vilnius University, Saulėtekio al. 7, LT-10257 Vilnius, Lithuania; raminta.batiuskaite@gmail.com (R.B.); rasa.burneikiene@bti.vu.lt (R.P.-B.); 2 Institute of Biochemistry, Life Sciences Center, Vilnius University, Saulėtekio al. 7, LT-10257 Vilnius, Lithuania; eugenijus.simoliunas@bchi.vu.lt (E.Š.); simonas.pajeda@gmail.com (S.P.); rolandas.meskys@bchi.vu.lt (R.M.)

**Keywords:** bacteriophage vB_EcoS_NBD2, tail tube protein, self-assembly, tubular structure, polytubes, stability, *Saccharomyces cerevisiae*, *Escherichia coli*

## Abstract

Nucleotides, peptides and proteins serve as a scaffold material for self-assembling nanostructures. In this study, the production of siphovirus vB_EcoS_NBD2 (NBD2) recombinant tail tube protein gp39 reached approximately 33% and 27% of the total cell protein level in *Escherichia coli* and *Saccharomyces cerevisiae* expression systems, respectively. A simple purification protocol allowed us to produce a recombinant gp39 protein with 85%–90% purity. The yield of gp39 was 2.9 ± 0.36 mg/g of wet *E. coli* cells and 0.85 ± 0.33 mg/g for *S. cerevisiae* cells. The recombinant gp39 self-assembled into well-ordered tubular structures (polytubes) in vivo in the absence of other phage proteins. The diameter of these structures was the same as the diameter of the tail of phage NBD2 (~12 nm). The length of these structures varied from 0.1 µm to >3.95 µm, which is 23-fold the normal NBD2 tail length. Stability analysis demonstrated that the polytubes could withstand various chemical and physical conditions. These polytubes show the potential to be used as a nanomaterial in various fields of science.

## 1. Introduction

Self-assembly is a spontaneous and specific interaction of molecular subunits into ordered shapes [1]. Peptides [2,3,4,5], proteins [6,7] and nucleotides [8,9,10] serve as a scaffold material for self-assembling nanostructures. A more complex type of self-assembling structures is virus-based nanoparticles (VNPs) [11,12,13,14,15,16]. The icosahedral nanostructures have been the most extensively produced and studied [17,18,19]. However, rod-shaped nanostructures lack variety as they are mostly derived from filamentous bacteriophages M13 and its relatives [20,21,22,23].

Self-assembling structures can be synthesized in more than 170 expression systems [24,25]. The generation of bacterial and plant VNPs is done using bacterial expression systems, mostly *E. coli* [26]. However, utilization of E. coli as a host is not always possible often due to recombinant protein misfolding and inclusion body formation [27]. Bacteria are not suitable for the synthesis of eukaryotic proteins due to their innate inability to introduce post-translational glycosylation modifications [28] and the presence of endotoxins in bacteria-derived preparations [26,29]. About 20% of reported VNPs have been produced in “endotoxin-free” yeast expression systems [26,30,31,32]. As a unicellular eukaryotic organism, yeast combine simple cultivation techniques and the capability to incorporate many post-translational modifications necessary for the production of biologically active or mammalian recombinant proteins [33]. Therefore, yeast-generated VNPs have been used as licensed vaccines against human papilloma [34] and hepatitis B viruses [35].

More than 5500 phages have been identified over the last five decades. About 95% of them are tailed bacteriophages and belong to three families of the *Caudovirales* order. The *Myoviridae* family consists of phages with long contractile tails, *Siphoviridae*—long non-contractile tails, and *Podoviridae*—short tails [36]. Tail assembly of siphoviruses is a strictly regulated process for which initiation complex is necessary [37,38,39]. The tail tube protein polymerizes as a stack of hexameric or unusual trimeric rings, such as in siphophages T5 [40] and phiCbK [41], around the tape measure protein forming long and flexible tails [39,42]. On the other hand, the recombinant tail tube protein from siphovirus SPP1 self-assembled into tubular structures of variable lengths in vitro without other phage proteins [43]. This indicates that very little is known about the assembly of tail tube proteins.

Various structural proteins of bacteriophages have been used to generate self-assembled nanostructures [43,44,45,46,47,48,49,50]. However, a little information regarding the self-assembly of phage tail tube proteins is available [41,43]. This study aims to contribute to this growing area of research by exploring the self-assembly mechanism of the tailed bacteriophage vB_EcoS_NBD2 (shortly called NBD2) [51]. The results of our study show that *E. coli*- or *S. cerevisiae*-synthesized recombinant tail tube protein gp39 is capable of self-assembling in vivo into long and flexible tubular structures in the absence of other viral proteins.

## 2. Materials and Methods

### 2.1. Strains

All cloning procedures were performed in E. coli DH10B (Invitrogen, Dublin, Ireland), DH5αF’ or GM119^−^ strains (laboratory strains from the Department of Eukaryote Gene Engineering, Institute of Biotechnology, Vilnius University). *E. coli* BL21 (DE3) (Novagene, Madison, WI, USA) and *S. cerevisiae* AH22-214 strains (laboratory strain from the Department of Eukaryote Gene Engineering) were used for heterologous protein synthesis.

### 2.2. Construction of an Expression Vector in Bacteria

DNA cloning was performed according to the standard molecular biology protocols [50]. Molecular mass standards, enzymes and kits for work with DNA were purchased from Thermo Fisher Scientific (Vilnius, Lithuania). Phage NBD2 gene *39* (Gene ID: 29079469) was amplified using NBD2 wild-type DNA as a matrix and NBD2_39_F 5′-CAAAGGAGTTTCAT**ATG**TCTCTTC-3′ and NBD2_39_R 5′-CTCTTGTTGGATCCAGTCGC-3′ primers (Metabion, Planegg, Germany). Primers included *Nde*I and *BamH*I recognition sites (underlined), and the protein translation initiation codon (shown in bold). The purified PCR product was cleaved with *Nde*I and *BamH*I, then inserted into the pET21a (Novagene, Madison, WI, USA) shuttle vector digested with the proper restriction endonucleases. The plasmid construct pET21a-NBD2-gp39 was maintained in *E. coli* DH10B cells, verified by DNA sequencing and used for transformation of *E. coli* BL21 (DE3) cells.

### 2.3. Construction of an Expression Vector in Yeast

A pET21a-NBD2-gp39 plasmid was used as a template for the construction of a pFX7-NBD2-gp39 vector for the synthesis in *S. cerevisiae*. The PCR was performed using Phusion High Fidelity DNA polymerase, NBD2_39_F 5′-TGTCTAGAACA**ATG**TCTCTTCCAAATGGTTC-3′ and NBD2_39_R 5′-AGTCTAGA**TTA**GTCAACTTCGCCCTGC-3′ primers (Metabion, Planegg, Germany), where *Xba*I recognition sites are underlined, and the protein translation initiation and termination codons are shown in bold. The obtained DNA fragments were digested with *Xba*I and ligated into an *Xba*I-linearized yeast expression vector pFX7 downstream of the hybrid GAL10-PYK1 promoter [52]. The plasmid construct pFX7-NBD2-gp39 was maintained in E. coli DH5αF’ cells, verified by DNA sequencing and used for transformation of *S. cerevisiae* cells.

### 2.4. Synthesis of Recombinant gp39 Protein in Bacteria and Yeast Cells

The synthesis of the gp39 protein was carried out in an *E. coli* BL21 (DE3) strain transformed with the plasmid pET21a-NBD2-gp39. Cell culture was grown in LB medium at 37 °C to an OD_600_ of 0.5, induced with 0.1 mM IPTG and incubated overnight at 20 °C. pFX7-NBD2-gp39 plasmid-transformed *S. cerevisiae* cells were grown in YEPD medium (1% yeast extract, 2% peptone, and 2% dextrose, supplemented with 5 mM formaldehyde) with shaking at 30 °C for 18–24 h. The synthesis of recombinant gp39 in yeast was induced by adding 3% galactose solution. Induced cells were grown for an additional 18–24 h with shaking as described previously [52].

### 2.5.Purification of Recombinant gp39 Protein

The yeast-produced recombinant gp39 protein was purified according to the previously described methodology [51,52] with some minor modifications. Five gram of wet induced yeast cells was resuspended in 10 mL of disruption buffer DB450 + Arg (10 mM Tris-HCl, 450 mM NaCl, 1 mM CaCl2, 0.01% TritonX-100, pH 7.2, 250 mM Arg, 2 mM PMSF). Then an equal volume of glass beads (0.5 mm diameter, Sigma Aldrich Co., St. Louis, MO, USA) was added and cells were disrupted by vortexing for 5 min at 4 °C. In parallel, induced bacteria cells were resuspended in DB450 + Arg buffer and disrupted by sonication. Cells were sonicated on ice, in 2.0 mL volume microcentrifuge tubes at 30% amplitude for 5 min of total ON time (30 s on/30 s off) by using the SonoPuls HD 2070 homogenizer (BANDELIN electronic GmbH and Co. KG, Berlin, Germany). The cell debris was separated by centrifugation at 9400× g for 15 min at 4 °C (Beckman Coulter Avanti J26 XP Centrifuge, Indianapolis, IN, USA). The majority of the recombinant gp39 protein was found in the soluble protein fractions using both expression systems. These fractions were collected and transferred onto 10 mL 40%/ 4 mL 30% (w/v) sucrose gradient in DB150 buffer (10 mM Tris-HCl, 150 mM NaCl, 1 mM CaCl2, 0.01% TritonX-100, pH 7.2). The proteins were sedimented by centrifugation at 140,000× g for 2 h at 4 °C (Beckman Coulter Optima L-90 K ultracentrifuge, rotor 60Ti or 70Ti, Indianapolis, IN, USA). The protein pellets containing recombinant gp39 were resuspended in DB150 buffer and analyzed by SDS-PAGE. The total protein concentration was measured with a spectrophotometer (NanoDrop 2000/2000c, Thermo Fisher Scientific, Wilmington, DE, USA). It allowed us to load the same amount of bacteria- and yeast-derived recombinant gp39 protein into SDS-PAGE. Gels were stained with Coomassie brilliant blue and the intensities of the desired protein bands were measured with ImageJ 1.50b software [53].

### 2.6. Transmission Electron Microscopy

The tail tube protein samples, except those used for protein stability analysis in different composition pH buffers or in urea, were suspended in phosphate buffer (100 mM NaCl, 80 mM Na_2_HPO_4_, 250 mM NaH_2_PO_4_, pH 7.4). Approximately 0.2–0.5 mg of the purified recombinant gp39 protein was placed onto 400-mesh carbon-coated copper grids (Agar Scientific, Stansted, UK). The sample was stained with 2% aqueous uranyl acetate solution (Reachim, Moscow, Russia) and analyzed with a Morgagni-268(D) electron microscope (FEI, Eindhoven, Netherlands).

### 2.7. Stability Analysis of Yeast- and Bacteria-Expressed Tail Tube Proteins

The tubular structure stability was analyzed using the purified recombinant gp39 protein samples in four approaches: (a) proteolysis; (b) incubation of gp39 for seven days in buffer containing 6 M urea; (c) heating the purified protein at 100 °C for 30 min; (d) incubation of gp39 protein in buffers of various pH as described earlier [33] with minor modifications. In the last approach, the recombinant gp39 protein was dialyzed against citrate, acetate, phosphate, Tris and carbonate buffers of pH 3.2–9.6 (Table 1), and each dialyzed protein sample was stored for seven days at 4 °C. To analyze the susceptibility of gp39 to proteolysis, the purified protein samples were treated with trypsin (Thermo Fisher Scientific, Vilnius, Lithuania) for 6 h at 37 °C. The protease was added to the protein sample to a final protease to protein ratio of 1:20 (w/w) according to manufacturer instructions. After each analysis, the recombinant gp39 protein was analyzed by SDS-PAGE and electron microscopy.

### 2.8. Bioinformatics Analysis

The bioinformatics analysis of the gp39 was performed using Transeq [54], Fasta-Nucleotide, Fasta-Protein, BLASTP and Clustal Omega [55]. The phylogenetic analysis was conducted using MEGA version 7 [53]. The protein information resource (PIR) server was used for calculating the predicted molecular mass of the recombinant protein [56]. The prediction of gp39 protein fold was conducted using the HHpred server [57,58,59].

## 3. Results

### 3.1. Bioinformatics Analysis

Based on the results of bioinformatics analysis, the tail tube protein of enterobacteria phage NBD2 encoded by gene *39* (Gene ID: 29079469) [51] had the closest identity (82%) to the tail tube protein of *Escherichia* phage vB_EcoS_ESCO4. Therefore, these two phages were assigned as unclassified siphoviruses within the subfamily *Tunavirinae*. The phylogenetic analysis revealed no close relationship between tail tube proteins from previously mentioned phages and the tail tube proteins, for which the structures are known (Supplementary Figure S1).

The amino acid sequence of gp39 protein corresponded to the fold of the two structures of bacteriophages, which was determined using the HHpred server. The residues 29 to 105 of gp39 protein were predicted to adopt the fold of the major tail protein, gpV, of bacteriophage lambda (PDB ref 2K4Q) with a probability of 99.62 (E-value, 4.1 × 10^−17^). The residues 31 to 177 of gp39 were aligned with the head-to-tail interface of bacteriophage SPP1 (PDB ref 5A21) with a probability of 98.85 (E-value, 7.3 × 10^−10^).

### 3.2. Synthesis and Purification of the Recombinant Tail Tube Protein gp39 in Bacteria and Yeast Cells

**Figure 1:**
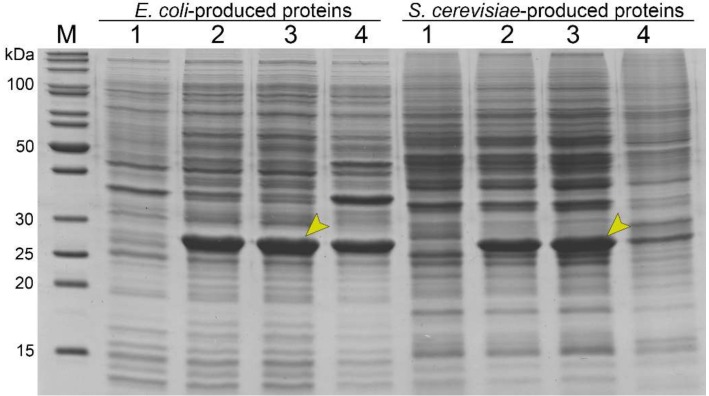
SDS-PAGE analysis of bacteria- and yeast-produced recombinant tail tube protein gp39. Approximately 35 µg of proteins were separated in a 14% SDS-PAGE gel in each lane and stained with Coomassie brilliant blue. M—page ruler pre-stained protein ladder (Thermo Fisher Scientific, Vilnius, Lithuania); lane 1—lysates of bacteria (pET21a) and yeast (pFX7) cells; lane 2—lysates of cells transformed with a corresponding plasmid (pET21a-NBD2-gp39 or pFX7-NBD2-gp39); lane 3—soluble fraction of transformed cells, where 28 kDa protein bands are indicated by the arrows; lane 4—insoluble proteins.

**Figure 2:**
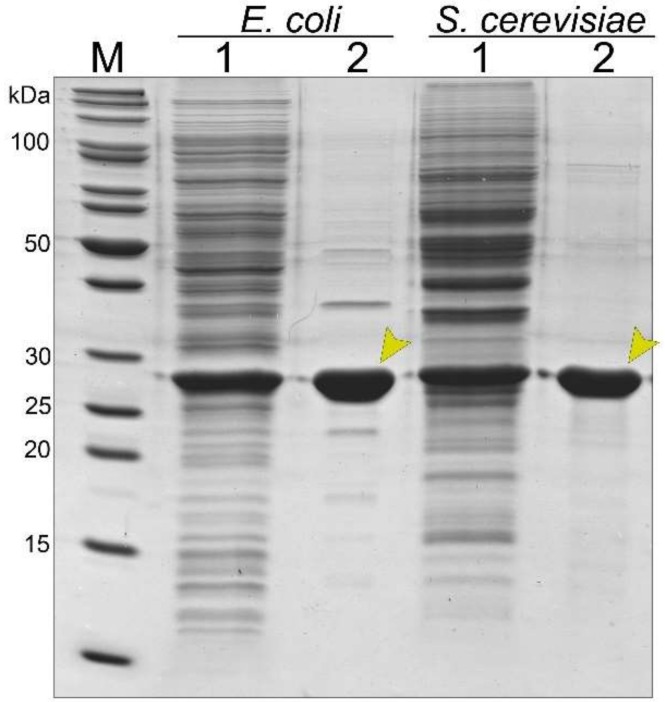
SDS-PAGE analysis of bacteria- and yeast-derived recombinant gp39 protein purification. In each lane, approximately 35 µg of proteins were separated. M—page ruler unstained protein ladder (Thermo Fisher Scientific, Vilnius, Lithuania); lane 1—soluble fraction of transformed cells with a plasmid (pET21a-NBD2-gp39 or pFX7-NBD2-gp39); lane 2—the purified recombinant gp39 protein is indicated by the arrows.

The recombinant gp39 protein was produced in *E. coli* and *S. cerevisiae* expression systems. The recombinant protein synthesis was analyzed by SDS-PAGE. A protein band of approximately 28 kDa was present when analyzing the lysates of *E. coli* and *S. cerevisiae* cells carrying pET21a-NBD2-gp39 or pFX7-NBD2-gp39 vectors, respectively (Figure 1, lanes 2). This band corresponded to the calculated molecular mass (~24 kDa) of gp39 of NBD2. In contrast, no additional band of the same molecular mass was observed when analyzing the lysates of empty plasmid-transformed cells (Figure 2, lanes 1). The recombinant gp39 protein constituted approximately 33% of the total cell proteins in cell lysate samples when produced in *E. coli* cells and 27% of the total cell proteins in *S. cerevisiae* cells, according to the analysis of the Coomassie brilliant blue-stained gel.

Bacteria and yeast cells were disrupted by sonication or mechanically with glass beads during recombinant protein purification. The soluble and insoluble proteins were separated by centrifugation. Bacteria-produced recombinant gp39 protein was found in both soluble and insoluble protein fractions, while yeast-produced tail tube protein was mostly found in the soluble protein fraction as evidenced by SDS-PAGE (Figure 1, lane 3).

The concentration of the recombinant gp39 protein in the soluble fraction was almost equal in both expression systems as evidenced by ImageJ software. Thus, the same volume of bacteria- and yeast-produced soluble proteins were transferred onto a sucrose gradient. The purity and quantity of target proteins were evaluated using SDS-PAGE and by ImageJ 1.50b software. Bacteria- and yeast-synthesized recombinant gp39 protein was estimated to be ~85% and ~90% pure, respectively (Figure 2). The yield of recombinant gp39 protein was 2.9 ± 0.36 mg/g of wet cells for *E. coli* and 0.85 ± 0.33 mg/g for S. cerevisiae (the data was collected from three independent purifications).

### 3.3. Electron Microscopy Analysis of Bacteria- and Yeast-Derived Tubular Structures

**Figure 3:**
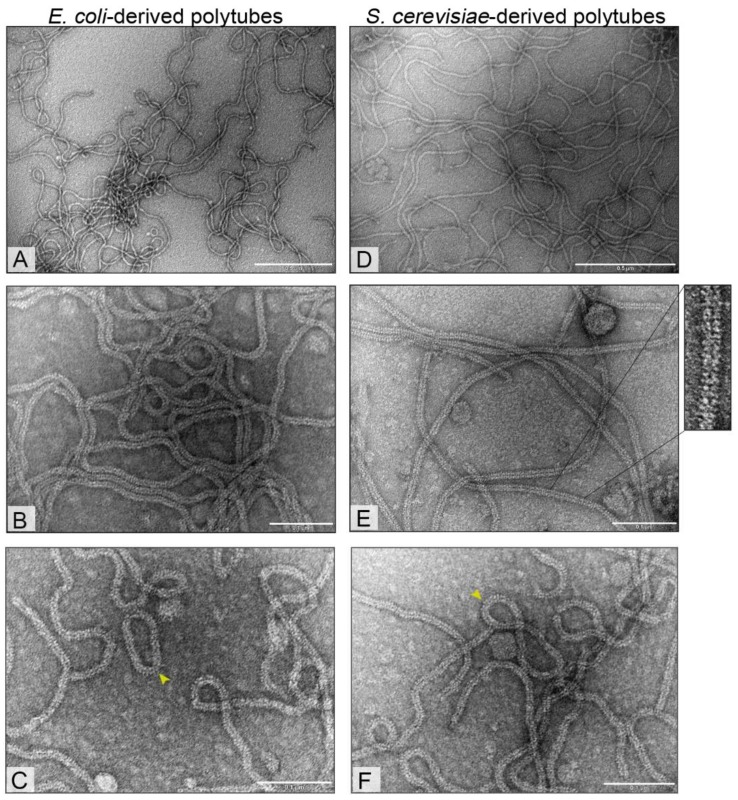
Electron micrographs of bacteria- (**A**–**C**) and yeast-derived (**D**–**F**) polytubes. Occasionally, the polytubes undergo end-to-end associations which results in closed circular structures (indicated by the arrows). The polytubes were suspended in phosphate buffer (pH 7.4) and analyzed with a Morgagni-268 (D) microscope (FEI, Eindhoven, Netherlands). In images (**A**,**D**) the scale bar indicates 0.5 µm, while in images (**B**,**C**,**E**,**F**) the scale bar is 0.1 µm.

Transmission electron microscopy (TEM) revealed the presence of extremely long and exceptionally flexible tubular structures (polytubes) formed by the recombinant tail tube protein found in cell-free extracts. *E. coli* and *S. cerevisiae*-derived particles were ~12 nm in width with variable lengths from 0.1 µm to >3.95 µm (Figure 3; Supplementary Figure S2). The tubular structures were formed from tail tube protein rings stacked onto each other (Supplementary Figure S3). Occasionally, the polytubes were shown to undergo end-to-end associations resulting in closed circular structures in both expression systems (Figure 3). Significant morphological differences between bacteria- and yeast-derived structures were not observed.

### 3.4. Stability of Bacteria- and Yeast-Derived Tubular Structures

To provide a comprehensive stability analysis of polytubes, the effects of boiling, proteolysis, incubation in the presence of urea or in buffers of different pH were evaluated. Protein degradation products or morphology changes of structures were monitored by SDS-PAGE and TEM analysis (Supplementary Figures S4–S7). No protein changes were observed in SDS-PAGE after boiling the samples for 30 minutes. However, the polytubes appeared to be less flexible and tend to aggregate with each other with almost no observed changes in structure length (Supplementary Figure S4). Incubation of the tubular structures in the presence of 6 M urea for seven days had no effect on the stability of recombinant proteins according to SDS-PAGE. In contrast, minor crack formations over the length of tubular structures after one day of incubation were observed by TEM. The number of cracks did not profoundly increase after prolonged treatment (Supplementary Figure S5).

Polytubes incubation for seven days in a wide range of different pH buffers (pH 3.2–9.6) resulted in partial degradation products only in buffers of pH 3.2–4.7. However, the partial degradation or any other detectable change in particle morphology was not observed in the TEM analysis (Supplementary Figure S6). Finally, the polytubes were incubated with trypsin protease, while hamster polyomavirus VP1 protein was used as a control [60]. The appearance of an additional molecular mass band of ~22 kDa demonstrated partial gp39 protein cleavage with no effect on polytube morphology (Supplementary Figure S7).

## 4. Discussion

We have demonstrated that *E. coli* and *S. cerevisiae* are suitable hosts for production of the recombinant tail tube protein gp39. Recombinant gp39 was found mostly in the soluble cell protein fractions in yeast. However, the same recombinant protein synthesized in bacteria was found in both the soluble and insoluble protein fractions. Various bacteria-derived recombinant proteins can often be found in an insoluble form [27]. For example, the majority of the recombinant tail tube protein of phage T5 was found in the insoluble fraction when produced in *E. coli* cells [61]. In contrast, *E. coli-*derived tail tube protein of siphovirus SPP1 was found in the soluble cell fraction [43].

The amount of recombinant gp39 protein in cell lysate samples was approximately 33% and 27% of the total cell proteins in E. coli and S. cerevisiae, respectively. The typical recombinant protein amount constitutes 20–50% of the total cell proteins in E. coli [62,63]. However, the synthesis of VP1 of murine polyomavirus reached only 2–3% of the total cell proteins in E. coli [64]. The amount of recombinant proteins usually constitute 1–5% of the total cell protein for S. cerevisiae [65] with some exceptions. Yeast-derived recombinant proteins sometimes can represent 10–15% of total cell proteins [66,67] or even reach up to 70% of the total cell protein level [68].

Inexpensive purification under native conditions (sucrose and arginine were used) allowed us to purify recombinant tail tube protein gp39 from both *E. coli* and *S. cerevisiae* hosts. The overall purity of bacteria- and yeast-derived recombinant gp39 samples in SDS-PAGE can be estimated as ~85% and ~90%, respectively. Production reached 2.9 ± 0.36 mg/g of wet cells for *E. coli* and 0.85 ± 0.33 mg/g for *S. cerevisiae*. Due to relatively fast bacteria growth and other differences between expression systems [69,70], it is common to purify 3.4-fold more recombinant gp39 protein from *E. coli* cells than from *S. cerevisiae* cells.

We report the first evidence that recombinant tail tube protein of siphovirus NBD2 self-assembled into polytubes in vivo in both *E. coli* and *S. cerevisiae* in the absence of other phage proteins. The polytubes were formed from structural tail tube proteins found in the soluble cell fraction in both expression systems. However, very little is known about the self-assembly of tail tube proteins of bacteriophages. Arnaud et al. demonstrated that *E. coli*-synthesized tail tube protein of T5 phage was detected in two forms. The soluble tail tube protein was monomeric, while insoluble tail tube protein in vivo assembled into polytubes up to several µm in length [61]. Langlois et al. demonstrated the self-assembly of *E. coli*-derived recombinant tail tube protein from siphovirus SPP1 into polytubes in the absence of other phage proteins. The assembly occurred in vitro during 3–10 days incubation forming structures with variable length [43]. Based on the published study, we predict that the length of the SPP1 polytubes was up to 500 nm. In another study, the sonication of phiCbK phage solution resulted in fragmentation of phage tails, which in vitro self-assembled into polytubes of variable length during several days of incubation [41]. Research shows that siphovirus tail assembly appears to be under stringent control [37,38] and uneven polytube length may result in the absence of phage regulatory proteins [39,71,72].

As evidenced by electron microscopy data, the polytubes of NBD2 are formed from a tail tube protein rings stacked onto each other. However, without comprehensive analysis, it is impossible to determine helical parameters of the NBD2 polytubes. To our knowledge, none of the crystal structures of the tail tube proteins from the subfamily *Tunaviridae* or their close relatives have been resolved to date. On the other hand, based on the structural homology of long tail extremities, *Siphoviridae* [73] and *Myoviridae* phages [74,75], bacterial Hcp-like proteins of type VI secretion system (T6SS) [76], and the tube proteins of R-, F-type pyocins [77,78] share a common ancestor. Mentioned tubular structures have a common helical organization. The tail tube and tail sheath of known myoviruses [74,79,80], most of the pyocins [77] and tail tube proteins of siphoviruses [43,81,82] typically form a stacked hexameric rings with the six-fold rotational symmetry.

*E. coli-* and *S. cerevisiae-*derived tubular structures with the diameter of ~12 nm and of varying length from 0.1 µm to >3.95 µm share a common morphology with native NBD2. Naturally, the NBD2 phage is characterized by having a non-contractile flexible tail ~12 nm in width and ~170 nm in length [51]. Remarkably, the length of self-assembled tubular structures was up to 23-fold the length of a normal NBD2 tail. To our knowledge, we demonstrate the longest in vivo self-assembled polytubes to date. Previously, in vitro self-assembly of tail tube fragments of phiCbK phage resulted in about eight-fold longer the normal tail length [41]. Additionally, the polytubes of NBD2 were found to be extremely flexible and occasionally associated end-to-end closed circular structures similar to phiCbK tail polymers [41].

The present study was designed to examine the structural stability of bacteria- and yeast-derived recombinant gp39 protein, as well as self-assembled polytubes. The polytubes tolerate various external factors since buffers (citrate, acetate, phosphate, Tris, carbonate) of different pH (3.2–9.6), detergents, boiling and trypsin protease seemed to have no or minor effects to the particle stability. To date, there are a few studies that have investigated the stability of self-assembled polytubes. For instance, the monomers of the tail tube protein of phage T5 are less stable in heat than the polymerized tubular structure [61]. The self-assembled polysheaths of phage T4, phiKZ and FV3 [47,48,49,50,76] were shown to remain stable under various external physical and chemical factors. While the self-assembly of urea-denatured sheath subunits of R-type pyocin [83] as well as type VI secretion system components was carried out [84,85], to our knowledge, the stability of these structures was not reported.

Finally, our study demonstrates that the tail tube protein of NBD2 in vivo self-assembled into extremely long and flexible polytubes in the absence of other phage proteins. We offer recombinant tail tube protein synthesis in different expression systems. *E. coli* allows for the generation of a higher yield of the recombinant gp39 protein. However, the inability to introduce post-translational glycosylation modifications and contamination with endotoxins or lipopolysaccharides limits the use in biological or medical applications [29]. Since yeasts are eukaryotic microorganisms, they are regarded as “endotoxin-free” systems capable of correctly folding eukaryotic proteins and introducing post-translational modifications [69,86]. The studies of self-assembled tubular structures from siphoviruses were performed in order to understand the polymerization properties, the tail morphology, and the tail tip-receptor recognition pathway, but not for the application purposes in biotechnology. Novel NBD2 polytubes could be used as flexible, extremely long platforms for foreign epitope display via genetic fusion or chemical modifications. The increased length-to-diameter ratio in tubular structures theoretically allows occupation of a higher number of binding sites on the cell surface resulting in a more efficient ligand-receptor binding for therapeutic [87], imaging [88], and targeting [89,90] purposes. Similarly, Wei and co-authors [91] observed the advantage of rod-shaped particles of 300 nm in length for induction of higher specific antibody titers over spherical VNPs. Nevertheless, the remarkable features of novel polytubes may be also useful for the fabrication of various nanowires [92].

## 5. Conclusions

This study has shown that the recombinant tail tube protein gp39 self-assembled into ordered polytubes in vivo, in the absence of other phage proteins, using either *E. coli* or *S. cerevisiae* expression systems. Simple and fast purification protocol allowed the production of recombinant proteins with 85‒90% purity. Finally, flexible and extremely long tubular structures were shown to remain intact under different chemical and physical conditions. Novel tubular structures could be used as flexible, extremely long platforms for foreign epitope display via genetic fusion or chemical modifications as well as for fabrication of nanowires.

## Figures and Tables

**Table 1:** The composition and pH of buffers used for recombinant gp39 protein stability analysis.

Composition of Buffers	pH
50 mM sodium citrate, 2 mM EDTA	3.2
50 mM sodium acetate, 2 mM EDTA	4.7
PBS, 2 mM EDTA	7.6
50 mM Tris, 2 mM EDTA	8.7
50 mM NaHCO_3_, 2 mM EDTA, 200 mM NaCl	9.6

## Supplementary Materials

The following are available online at https://www.mdpi.com/1999-4915/11/3/208/s1, Figure S1: Neighbor-joining tree analysis based on the alignment of the amino acid sequences of the tail tube proteins; Figure S2: Electron micrograph of an extremely long self-assembled polytube; Figure S3: Electron micrograph of polytubes formed from a tail tube protein rings stacked onto each other; Figure S4: Thermal stability of tubular structures; Figure S5: The stability analysis of (A) E. coli- and (B) S. cerevisiae-derived polytubes in buffer containing 6 M urea; Figure S6: The stability of (A) E. coli- and (B) S. cerevisiae-derived polytubes in pH 3.2–9.6; Figure S7: The effect of trypsin protease on polytube stability.
