# Supplementary material for: Self-Assembly of Tail Tube Protein of Bacteriophage vB_EcoS_NBD2 into Extremely Long Polytubes in E. coli and S. cerevisiae
Source: Viruses. 2019 Mar 1;11(3):208. doi: 10.3390/v11030208 (PMC6466441; doi:10.3390/v11030208)
Supplement: Supplementary file 1 [file viruses-11-00208-s001.pdf]

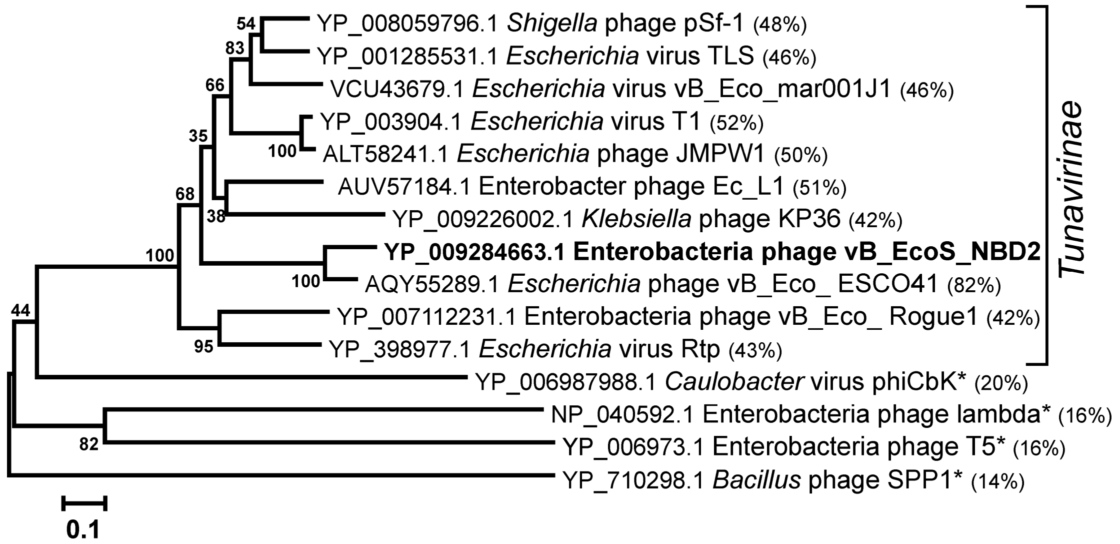

Supplementary Figure S1. Neighbor-joining tree analysis based on the alignment of the amino acid sequences of the tail tube proteins. The phylogenetic analysis was conducted using MEGA version 7. The percentage of replicate trees in which the associated taxa clustered together in the bootstrap test is shown next to the branches. The identity in amino acid level (%) between gp39 of NBD2 and tail tube proteins of phages is indicated in brackets. * – tail tube proteins with known structures [1–5].

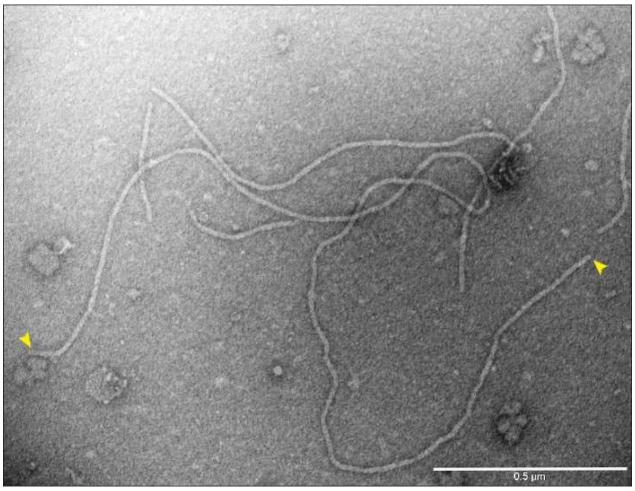

Supplementary Figure S2. Electron micrograph of an extremely long self-assembled polytube. The purified polytubes were suspended in phosphate buffer (pH 7.4) and analyzed with a Morgagni-268(D) microscope (FEI, Eindhoven, Netherlands). The arrows indicate two ends of the flexible polytube (3.95 µm in length). The scale bar = 0.5 µm.

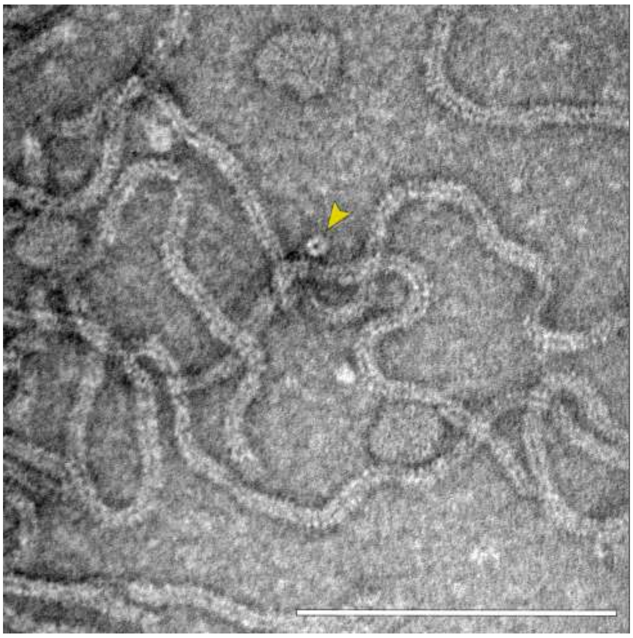

Supplementary Figure S3. Electron micrograph of polytubes formed from a tail tube protein rings stacked onto each other. The protein sample was suspended in phosphate buffer (pH 7.4) and analyzed with a Morgagni-268(D) microscope (FEI, Eindhoven, Netherlands). The arrow indicates a single ring of associated recombinant tail tube proteins. The scale bar = 0.2 μm.

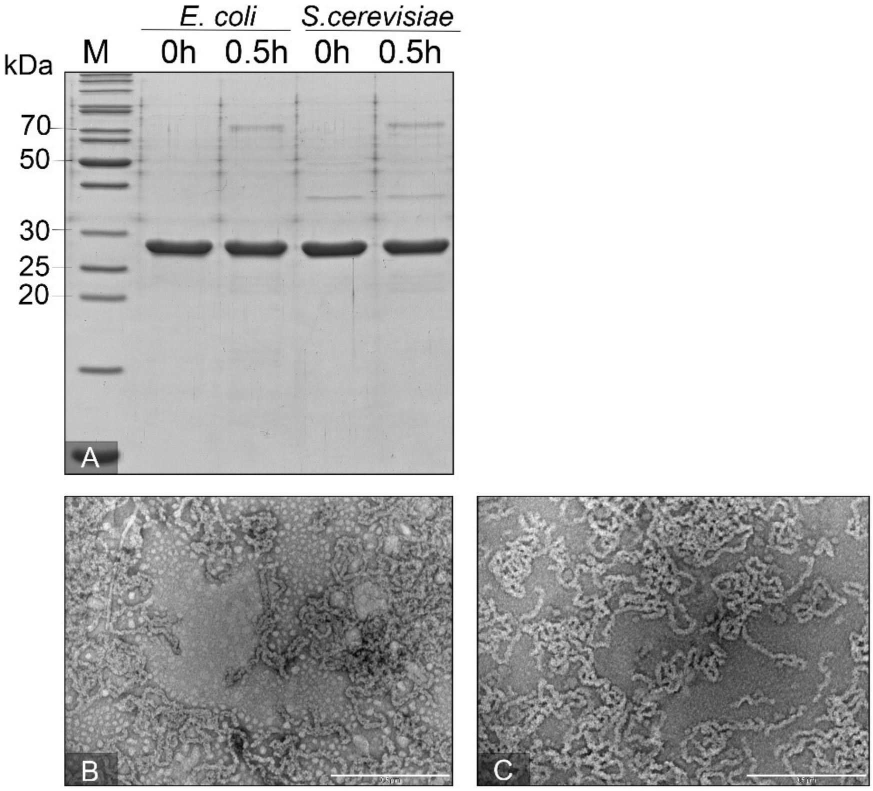

Supplementary Figure S4. Thermal stability of tubular structures. **(A)** The SDS-PAGE of *E. coli*- and *S. cerevisiae*-derived tubular structures. M – Page Ruler Unstained Protein Ladder (Thermo Fisher Scientific, Vilnius, Lithuania); the boiling time is indicated above each lane. Electron micrographs of **(B)** bacteria- and **(C)** yeast-derived tubular structures were taken after boiling them for 0.5 h. The protein samples were suspended in phosphate buffer (pH 7.4). The magnification 56,000×, scale bars = 0.5 μm.

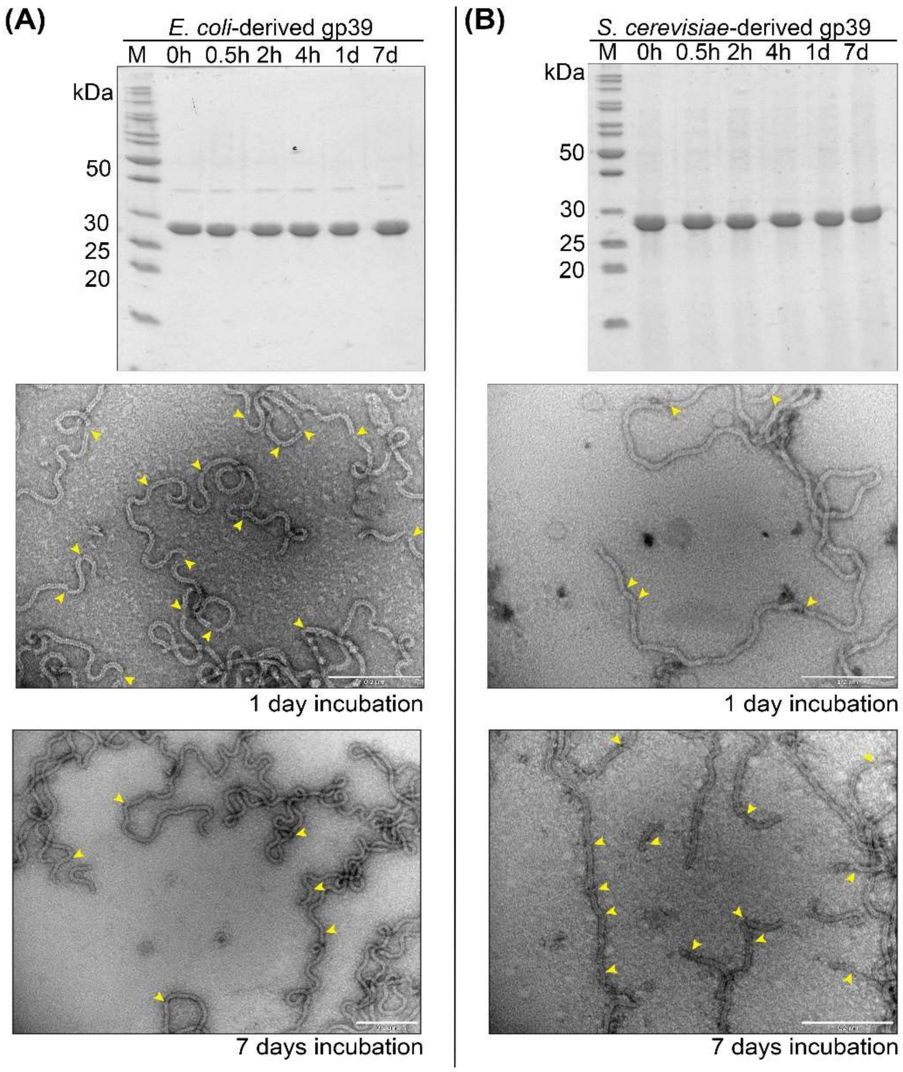

Supplementary Figure S5. The stability analysis of **(A)** *E. coli*- and **(B)** *S. cerevisiae*-derived polytubes in buffer containing 6 M urea. M – Page Ruler Unstained Protein Ladder (Thermo Fisher Scientific, Vilnius, Lithuania); the time points of incubation in 6 M urea buffer are indicated above each lane. The cracks along the polytubes started to appear after one day of incubation (marked by the arrows), while the recombinant protein remained intact. The magnification 140,000×, scale bars = 0.2 μm.

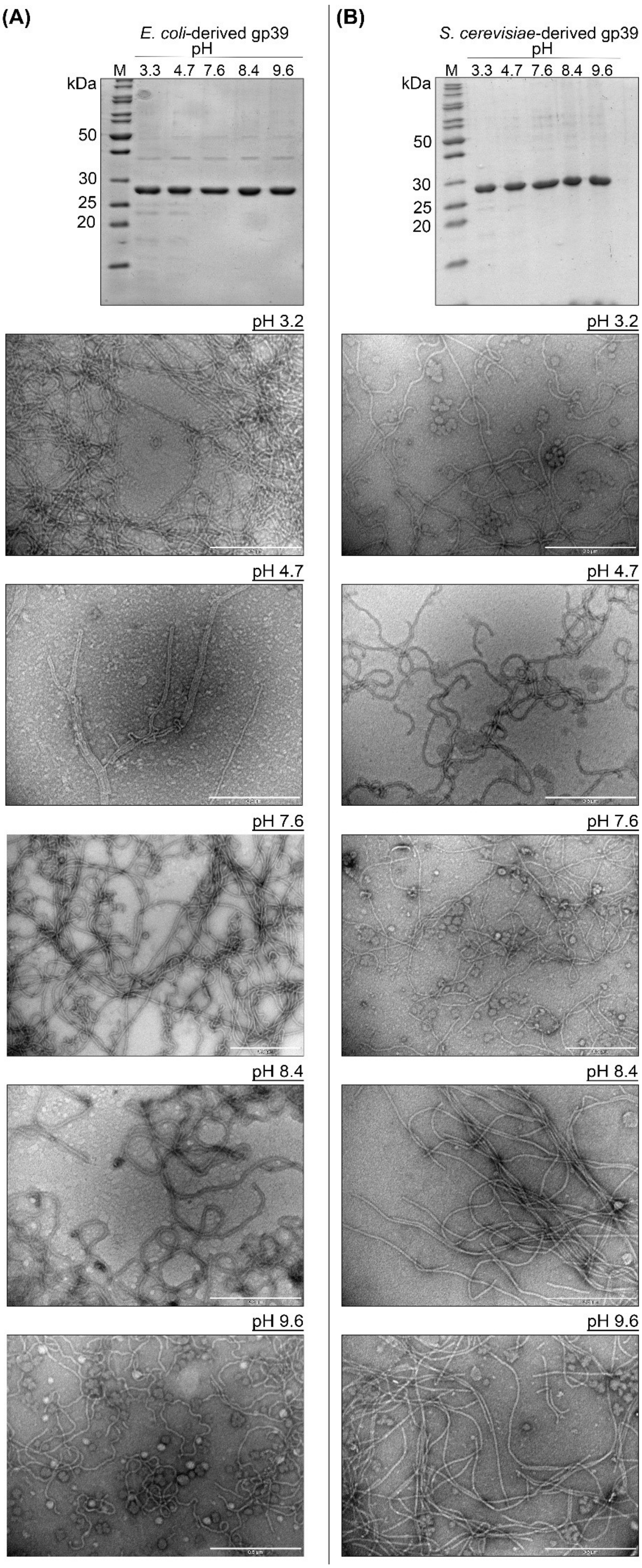
(A)
E. coli-derived gp39
pH
M 3.3 4.7 7.6 8.4 9.6
kDa
50
30
25
20
pH 3.2
pH 4.7
pH 7.6
pH 8.4
pH 9.6
(B)
S. cerevisiae-derived gp39
pH
M 3.3 4.7 7.6 8.4 9.6
kDa
50
30
25
20
pH 3.2
pH 4.7
pH 7.6
pH 8.4
pH 9.6

Supplementary Figure S6. The stability of **(A)** *E. coli*- and **(B)** *S. cerevisiae*-derived polytubes in pH 3.2–9.6. M – Page Ruler Unstained Protein Ladder (Thermo Fisher Scientific, Vilnius, Lithuania); the different pH buffers are indicated above each lane as follows: citrate, acetate, phosphate, Tris and carbonate. Incubation in pH 3.2 and 4.7 buffers resulted in the appearance of weak intensity protein bands of <28 kDa with no effect on polytube morphology. The magnification 56,000x, scale bars = 0.2 μm.

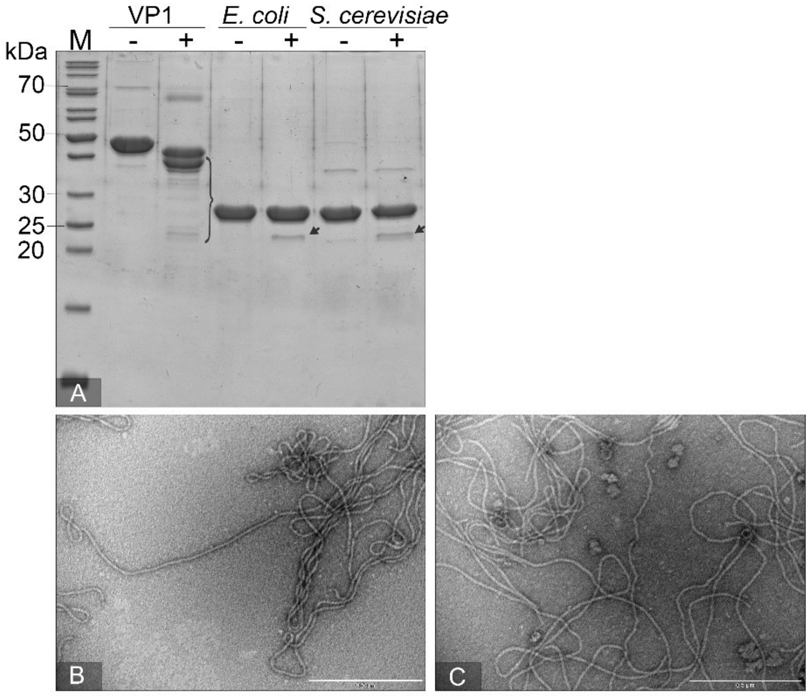

Supplementary Figure S7. The effect of trypsin protease on polytube stability. **(A)** SDS-PAGE analysis of hamster polyomavirus VP1 protein, *E. coli*- and *S. cerevisiae*-derived polytubes. M – Page Ruler Unstained Protein Ladder (Thermo Fisher Scientific, Vilnius, Lithuania); lanes "-" indicate native recombinant proteins, while "+" represent trypsin proteolysis products. Trypsin protease has cleaved hamster polyomavirus VP1 into several fragments of molecular masses <48 kDa (indicated by the brackets). Incubation of recombinant gp39 protein with trypsin protease in phosphate buffer (pH 7.4) resulted in the appearance of smaller molecular mass fragments of approximately 24 kDa in size (indicated by the arrows). The stability of **(B)** bacteria- and **(C)** yeast-derived polytubes is evidenced by the TEM analysis. The magnification 56,000×, scale bars = 0.5 μm.

References

1. Leonard, K. R.; Kleinschmidt, A. K.; Lake, J. A. Caulobacter crescentus Bacteriophage phiCbK: structure and in vitro self-assembly of the tail. *J. Mol. Biol.* **1973**, *81*, 349–365, doi:10.1016/0022-2836(73)90146-0.

2. Langlois, C.; Cukkemane, A.; Auzat, I.; Chagot, B.; Gilquin, B.; Ignatiou, A.; Petitpas, I.; Kasotakis, E.; Paternostre, M.; White, H. E.; Orlova, E. V; Baldus, M.; Tavares, P.; Zinn-Justin, S. Bacteriophage SPP1 tail tube protein self-assembles into β-structure-rich tubes. *J. Biol. Chem.* **2015**, *290*, 3836–3849, doi:10.1074/jbc.M114.613166.

3. Katsura, I. Tail assembly and injection. In *Lambda II*; Hendrix, R., Roberts, J., Stahl, F., Weisberg, R., Eds.; Cold Spring Harbor, Plainview, NY, USA **1983**; pp. 331–346, doi:10.1101/087969150.13.331.

4. Pell, L. G.; Kanelis, V.; Donaldson, L. W.; Howell, P. L.; Davidson, A. R. The phage lambda major tail protein structure reveals a common evolution for long-tailed phages and the type VI bacterial secretion system. *Proc. Natl. Acad. Sci. U S A* **2009**, *106*, 4160–4165, doi:10.1073/pnas.0900044106.

5. Arnaud, C. A.; Effantin, G.; Vives, C.; Engilberge, S.; Bacia, M.; Boulanger, P.; Girard, E.; Schoehn, G.; Breyton, C. Bacteriophage T5 tail tube structure suggests a trigger mechanism for Siphoviridae DNA ejection. *Nat. Commun.* **2017**, *8*, 1953, doi:10.1038/s41467-017-02049-3.
